# Two-Year Experience of Orthotopic Liver Transplantation in Afzalipoor Hospital, Kerman, Southeastern Iran

**Published:** 2012-08-01

**Authors:** M. Dehghani, B. Poorseidi, H. Sattari, S. Nikeghbalian, M. J. Zahedi, S. M. Seyyed-Mirzaei, M. Shafiei, M. Vahedian, S. A. Malek-Hosseini

**Affiliations:** 1 *Organ Transplant Research Center, Afzalipoor Hospital, Kerman University of Medical Sciences, Kerman, Iran*; 2 *Organ Transplant Research Center, Nemazee Hospital, Shiraz University of Medical Sciences, Shiraz, Iran*

**Keywords:** Liver transplantation, Liver disease, Iran

## Abstract

Background: End-stage liver diseases are common in Iran. The only therapeutic option for these patients is liver transplantation.

Objective: To present our 2-year experience of liver transplantations in Afzalipoor Hospital, Kerman, southeastern Iran.

Methods: From November 2009 to September 2011, 12 patients underwent orthotopic liver transplantation in our center. Their data including demographics, indications for transplantation, MELD scores, post-operative complications and their management were collected.

Results: Patients (7 women and 5 men) aged between 14 and 55 years. Indications for the transplantation included HBV infection (n=5), cryptogenic cirrhosis (n=2), Wilson’s disease, alcoholism (n=1), HCV infection (n=1), Budd-Chiari syndrome (n=1), and autoimmune hepatitis (n=1). MELD score of patients ranged from 16 to 30. All patients received tacrolimus, mycophenolate mofetile and corticosteroid, postoperatively. 2 patients died of pulmonary and intra-abdominal infections with resultant to multiple organ failure. Nonfunctioning of transplanted liver and ongoing bleeding resulted in death in another patients. 9 patients are well doing and have excellent liver functions.

Conclusion: We had relatively successful results in our experience of orthotopic liver transplantation. Vicinity of our center to Shiraz Transplant Center would be an important factor in this success.

## INTRODUCTION

Liver transplantation is the treatment of choice for many patients with end-stage liver disease; it is performed on a routine basis in most major centers throughout the world. After a series of initial failures, the first successful human liver transplantation took place in 1967 by Thomas E. Starzl in Denver, USA. In Iran, the first orthotopic liver transplantation was performed on May 4, 1993 by Seyed-Ali Malek-Hosseini in Nemazee Hospital, Shiraz. Soon, the center became the most important liver transplant center in the region.

End-stage liver disease and its complications are relatively common in Iran and creating another active transplant center seems necessary. Kerman University of Medical Sciences created a new center for liver transplant. Herein, we present our two-year results of liver transplantation in Afzalipoor Hospital, Kerman, southeastern Iran.

## PATIENTS AND METHODS

From November 2009 to October 2011, 50 patients with end-stage liver disease whom were referred by primary care physicians to our center, were evaluated for receiving a liver transplant. Patients were examined for the procedure by an expert committee consisted of a transplant surgeon, a hepatologist, a transplant anesthesiologist, a transplant coordinator and a trained nurse. The evaluation was mainly emphasized on anatomic suitability, *i.e.*, patency and size of portal vein, confirmation of the diagnosis, and the current status of the patient—progression of hepatic disease, a search for other major systemic disease, any associated vitamin and nutritional deficiencies and family situation.

Candidates for the procedure were selected mainly based on their MELD score and the criteria for decompensating liver failure. We did not include patients with primary malignancies because of organ shortage. Matching was done by size of the transplant and blood group.

Piggy-back without veno-veno bypass technique was used for all patients. The immunosuppression regimen used included tacrolimus and mycophenolate mofetile. An intravenous bolus of steroid—usually starting at 1000 mg of methylprednisolone—was given for three days followed by 20 mg/day oral prednisone. In one patient, tacrolimus, for its severe nephrotoxic side effects, was switched to sirolimus.

All patients with HBV received immunoglobulin to maintain an antibody titer of >500 IU/mL during the first month of transplantation; thereafter, the level was kept above 300 IU/mL.

We did not check the blood levels of tacrolimus; however, in recipients with transplant rejection the dose was increased; for those with toxic effects of the drug, the dose was decreased.

## RESULTS

Of 50 patients evaluated for liver transplantation, 15 were found ineligible, mainly because of disease of other organ systems that might have deleterious effect on the transplant (*i.e.*, severe primary renal disease or severe hepatogenic cyanosis). Of the remaining patients, eight died prior to liver transplantation; 12 underwent the transplantation, and 15 were in the waiting list. The mean waiting period from the time of evaluation to transplantation was three months.

The 12 recipients aged between 14 and 55 years. Indications for the transplantation included HBV infection (n=5), cryptogenic cirrhosis (n=2), Wilson’s disease, alcoholism (n=1), HCV infection (n=1), Budd-Chiari syndrome (n=1), and autoimmune hepatitis (n=1). Nine of 12 patients have survived 2 to 24 months of surgery; the graft function in all survivors was excellent.

All three deaths occurred within two months of transplantation. One patient with HBV liver disease died of severe pulmonary infection three weeks after operation, yet her transplanted liver function was good. Another death was in a 35-year-old man with HBV liver cirrhosis, severe intraperitoneal infection and severe malnutrition. Biliary duct anastomosis (duct to duct) was disrupted. This patient died two months after transplantation due to multiple organ failure. The last mortality was in a 55-year-old patient with alcoholic liver cirrhosis who died of nonfunctioning transplanted liver within 24 hours after transplantation.

In all patients bile duct reconstruction was done by duct to duct anastomosis without using a T-tube or stent. Two patients developed stricture at the site of bile duct anastomosis within the first month of transplantation; Roux-en-Y hepaticojejunostomy resolved the problem.

Preservation injury was developed in two patients; it was managed conservatively. One of the patients who had HCV, developed deep venous thrombosis in the upper extremity and thrombosis of the radial artery with dry gangrene of distal phalanx of the fifth finger; the patient was treated with anticoagulant therapy. New onset diabetes mellitus was another complication developed in two patients; it was treated with insulin.

## DISCUSSION

End-stage liver disease and its complications are common in Iran, but the only active center for managing of these patients is in Shiraz—Shiraz Transplant Center. The need for another active liver transplant center in Iran is therefore obvious.

Liver transplantation is a complex procedure. Kerman Liver Transplant Center performed the first liver transplantation in November 2009 under auspices of Shiraz transplant team. Until October 2011, 12 whole organ liver transplant from deceased donors were done at Afzalipoor Hospital, Kerman. The results of this primary effort are comparable with those of other approved centers. In two patients, portal vein was obstructed by an old thrombus; after thrombectomy successful liver transplantation was done. Now, portal vein thrombosis is not a contraindicaton for the operation.

The Achilles’ heel of the transplanted liver is bile duct reconstruction. Biliary complications are among the most common complications and in our series, bile duct stricture developed in two patients; another patient developed bile duct disruption with resultant severe intra-abdominal infection. Despite all efforts made, unfortunately this patient died.

Organ shortage is another potential important problem for organ transplantation that resulted in dying of eight patients in the waiting list. An intensive team effort including social services, is absolutely necessary for success of the procedure.

We believe that any new active liver transplant program in Iran should be linked with pioneering role of Shiraz Center. Solid organ transplant center of Kerman is rapidly developing due to increasing of organ donation from deceased donors, increasing experience of its transplant team and collaboration of effective social services with this new center.
